# Psychometric evaluation of the cardiac rehabilitation adherence scale in patients with coronary heart disease: an observational study

**DOI:** 10.3389/fcvm.2025.1641392

**Published:** 2025-09-11

**Authors:** Zhenshuai Yao, Xiaohui Wen, Juping Yu, Xiaofang Zhu, Jin Wang, Pingping He, Xinping Ouyang

**Affiliations:** ^1^ The Aging Health Research Center, School of Nursing, Medical Center, Hunan Normal University, Changsha, China; ^2^ School of Nursing, Hengyang Medical School, University of South China, Hengyang, China; ^3^ Nursing Department, Fudan University Shanghai Cancer Center Fudan University Cancer Institute, Shanghai, China; ^4^ The Faculty of Life Sciences and Education, University of South Wales, Pontypridd, United Kingdom

**Keywords:** coronary disease, cardiac rehabilitation, observational study, patient compliance, psychometrics

## Abstract

**Background:**

Cardiac rehabilitation has showed the potential to improve health outcomes of patients with coronary heart disease. However, the adherence of patients participating in cardiac rehabilitation is unsatisfactory due to some barriers. The quantitative instrument for measuring cardiac rehabilitation adherence is scarce. Hence, the purpose of this study was to develop a scientific tool and assess its psychometric properties in patients with coronary heart disease

**Material and Methods:**

The psychometric properties of the revised scale were tested with 509 patients. Item analysis was conducted to evaluate the discrimination and homogeneity of the scale. Content validity was evaluated by content validity index and Exploratory factor analyses and confirmatory factor analyses were used to examine the factor structure of the scale. Reliability was evaluated by Cronbach's coefficients and split-half reliability coefficients.

**Results:**

A scale covering five dimensions and thirty-three items was developed for evaluating cardiac rehabilitation adherence. The content validity index of the scale was 0.96. In exploratory factor analysis, a five-factor structure model was confirmed, explaining 71.255% of the total variation. In confirmatory factor analysis, the five- factor structure was supported by appropriate fitting indexes. In terms of reliability, the Cronbach's *α* coefficient of the scale was 0.909 and the spilt-half reliability coefficient of the scale was 0.765.

**Conclusion:**

The newly developed self-completion scale is reliable and valid. It appears to be a sound instrument for nurses and a broader range of healthcare professionals to effectively evaluate the cardiac rehabilitation adherence.

## 1 Introduction

Coronary heart disease (CHD) is a chronic illness in which the epicardial coronary artery becomes clogged or interrupted due to atherosclerotic plaque deposition, resulting in an inadequate blood flow to the myocardial (1). As the most prevalent type of cardiovascular diseases, CHD is the leading cause of morbidity and mortality worldwide and has become a major global healthcare burden (2). Although the mortality rate of CHD has decreased over the last four decades, it still accounts for approximately one-third of deaths in individuals over 35 years old (3). In China, its prevalence has increased rapidly in recent years as numerous risk factors have emerged including unhealthy lifestyle, obesity and air pollution, with about 11.39 million people suffering from the condition (4). Moreover, the mortality rate of CHD will continue to increase especially in developing countries (5). CHD is associated with detrimental consequences, including decreased functional capacity, increased stress and depression, compromised quality of life, and fear of recurrent cardiac events or mortality which are often reported by patients (6, 7). Hence, CHD has become a serious public health problem.

With the growing number of individuals living longer with CHD, the provision of accessible and effective health services for managing CHD becomes increasingly critical. Cardiac rehabilitation is widely recognized as a key component in the secondary prevention of coronary heart disease with the purpose of enhancing patients' quality of life and reducing disabilities and mortalities (8). It is a structured program that consists of medication therapy, dietary education, physical activity counselling, psychosocial support, and tobacco counselling (9). Cardiac rehabilitation has showed the potential to enhance health outcomes and accelerate recovery in patients with CHD (10). It is reported that participation in cardiac rehabilitation programs can lower the risk of cardiac events, reduce cardiac-related mortality by 26% and hospital re-admission by 31% (11, 12). Therefore, cardiac rehabilitation brings numerous benefits for patients with cardiovascular diseases.

However, these benefits depend on long-term and high adherence to cardiac rehabilitation. Unfortunately, participation in cardiac rehabilitation programs is far from optimal in many countries (13). For instance, in a US study, only 44% of the qualified patients participated in a cardiac rehabilitation program and 30% of the participants dropped out during the study (14). Summer et al. followed up 288,123 British patients participating in cardiac rehabilitation programs and found only 13% of the patients completed cardiac rehabilitation for more than 8 weeks (15). Many factors may contribute to non-adherence to cardiac rehabilitation, such as geographic inaccessibility, inconvenient transportation, competing priorities or demands and the expensive cost of rehabilitation from patients' angles (16). Moreover, lack of professional guidance from healthcare providers and limited rehabilitation infrastructure also constitute barriers in the implementation of practice (17, 18). Studies have proved that high participation rate and better compliance in cardiac rehabilitation programs are beneficial for patient recovery and functional outcomes in patients with CHD (19). Therefore, there is a need for a comprehensive and scientific evaluation of cardiac rehabilitation adherence to identify their behaviors and enhance the participation rate, which is crucial for improving clinical outcomes and patients' quality of life.

Currently, there is a lack of uniform and recognized criterion for assessing cardiac rehabilitation adherence for patients with coronary heart disease. In existing literature, a series of relevant scales were developed and validated to focus on assessing cardiac rehabilitation preferences and barriers such as the cardiac rehabilitation inventory, the cardiac rehabilitation preference form, the cardiac rehabilitation enrollment obstacles scale and the information needs in cardiac rehabilitation scale (20–23). However, none of these scales are suitable for assessing adherence to cardiac rehabilitation as such evaluation indicators are insufficient and fail to fully reflect cardiac rehabilitation adherence, which are not suitable for directly assessing behaviors of patients participating these programs. Moreover, the measurement indexes and their calculation formulas used in existing tools are often inconsistent, which limits the credibility and comparability of research results and hinders the promotion and application of these scales. Given the importance of cardiac rehabilitation, it is essential to create a reliable instrument for evaluating adherence to cardiac rehabilitation in patients with coronary heart disease to fill the gap in the research. Such a scale would help health professionals assess the adherence behavior of patients participating in cardiac rehabilitation programs and identify the level of their behaviors in a direct way. Therefore, the present study aimed to develop a cardiac rehabilitation scale and evaluate its psychometric properties among patients with coronary heart disease.

## 2 Material and methods

### 2.1 Study design and participants

**Figure 1:**
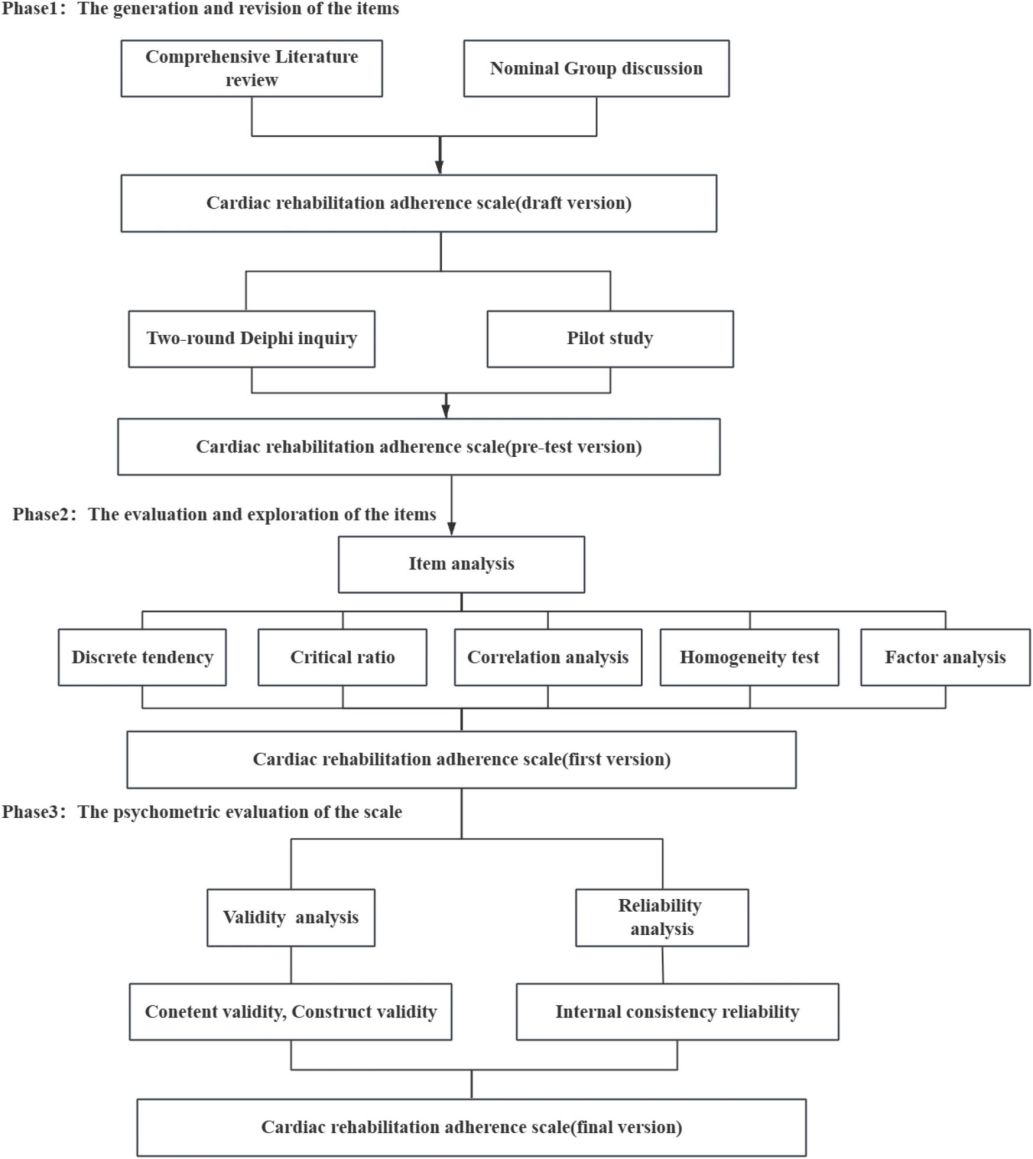
The development procedure of the cardiac rehabilitation scale.

This study used a cross- sectional methodological design. This scale development consisted of three stages:(1) item generation and revision (2) item evaluation and exploration (3) psychometric evaluation of the scale. The development process of the scale is depicted in Figure 1. This study adherently followed the guideline for strengthening the reporting of observational studies in epidemiology (STROBE) (24).

The research was carried out from March to September 2022. A convenience sampling method was employed to recruit patients with coronary heart disease from the Department of Cardiology at three tertiary hospitals in Hengyang, China. Eligibility criteria for participation in this phase included patients who met the WHO diagnostic criteria for coronary heart disease (25), possessed clear consciousness, were at least 18 years of age, provided informed consent, and had not undergone cardiac rehabilitation within the past year. Patients were excluded based on the presence of serious complications, including malignant tumors or severe organ failure, a classification of IV cardiac function according to the New York Heart Association (NYHA), or severe mental and psychological disorders that impaired normal communication abilities.

Six members of the research team at the three hospitals were tasked with recruiting potential participants. Two research assistants administered the questionnaires to patients directly. Prior to data collection, team members underwent systematic and organized training to ensure the provision of prompt guidance and assistance to participants. Based on the general rule of factor analysis, the sample size should generally be 5–10 times greater than the number of items on a questionnaire (26). The scale consisted of 35 items, with an anticipated sample size ranging from 175 to 350 participants. Considering the 20.0% of sample loss rate, 210–420 participants were required. A larger sample size over 420 is more preferable. A total of 509 patients with coronary heart disease were enrolled in this study eventually. Phase 1 did not include participants. This sample (*n* = 509) was utilized in both the second and third phases. During the third phase of factor analysis, samples were randomly divided into two groups: one for exploratory factor analysis (*n* = 254) and the other for confirmatory factor analysis (*n* = 255).

### 2.2 Data collection instruments

#### 2.2.1 Demographic information questionnaire

Participants' characteristics were captured using a self-compiled demographic information questionnaire, which included questions about gender, age, education level, monthly income, occupation, and marital status.

#### 2.2.2 Initial cardiac rehabilitation adherence scale in patients with CHD

The scale comprised questions regarding adherence to exercise, medication, risk factor management, nutrition management, and psychological management, encompassing 5 primary indicators and 35 secondary indicators. The available responses for each segment of the scale are: ①= yes, ②= no. The scale's total score ranges from 35 to 70, with higher scores indicating improved adherence to cardiac rehabilitation. Forward scoring questions were assigned points as follows: “Yes” received 2 points, while “No” received 1 point. In contrast, reverse scoring questions were assigned points such that “Yes” received 1 point and “No” received 2 points. Four items were scored in reverse, specifically item 7 in Dimension A (exercise adherence) and items 3, 4, and 5 in Dimension B (medication adherence).

### 2.3 Procedures

#### 2.3.1 Phase1: the generation and revision of the items

##### 2.3.1.1 Literature review

The review process was conducted in national and international databases including PubMed, Wed of science, Cochrane, CNKI, Wanfang, WeiPu and Chinese Biomedical literature database (CBM) were used to identify relevant literature about adherence behavior in cardiac rehabilitation published between 2012 and 2022.The search strategy for each database employed a combination of Mesh terms and free text, which is described in Supplementary Appendix S1. In addition, the reference lists from included studies were manually searched to ensure that all relevant literature were retrieved. Specific literature search strategy is exemplified by PubMed, as illustrated in Table 1.

**Table 1:** The search strategy of pubMed.

Step	Strategy
#1	“Coronary disease”[Mesh]OR “Coronary heart disease”[Title/Abstract]OR “myocardial ischemia”[Title/Abstract] OR “acute coronary syndrome”[Title/Abstract]OR “myocardial infarction”[Title/Abstract]OR “angina pectoris” [Title/Abstract] OR “percutaneous coronary intervention”[Title/Abstract]OR “percutaneous transluminal coronary angioplast” [Title/Abstract] OR “coronary artery bypass” [Title/Abstract]
#2	“Cardiac rehabilitation”[Mesh]OR“Cardiac rehabilitations *” [Title/Abstract]OR “Cardiovascular rehabilitation*”[Title/Abstract]OR “Rehabilitation *”[Title/Abstract]
#3	“Adherence”[Title/Abstract] “Compliance” [Title/Abstract] OR“Persistence”[Title/Abstract]
#4	#1 AND #2 AND #3

##### 2.3.2.2 Delphi expert consultation

This research employed a two-round Delphi method to evaluate the content validity of the scale. The Delphi survey aims to achieve consensus on a particular topic and is commonly utilized across various disciplines via an interactive process involving multiple rounds and questionnaires (27, 28). This approach is now a widely recognized method for the development of clinical measurements and has demonstrated reliability in developing new concepts and establishing consensus across various subject areas (29, 30).

The selection of experts is essential in the consensus-building process. Twenty-two specialists in chronic disease prevention and management, cardiovascular disease, nutrition and health, cardiac rehabilitation, and psychology were invited to participate in the Delphi expert consultation. The inclusion criteria required members to possess intermediate professional titles or higher, a minimum of 10 years of relevant work experience, and a bachelor's degree or higher. The Delphi panelists consented to participate in and complete two rounds of inquiry.

The two-round Delphi inquiry was conducted by distributing and collecting the inquiry questionnaire through Wechat, a widely used Chinese social media platform, and email in March and May 2022. The recovery rate of the question was utilized to indicate the level of enthusiasm among experts. The authority coefficient (Cr) represents the mean of familiarity with the field and the index judgment criteria. A Cr value exceeding 0.75 signifies a reliable consultation rate. Increased levels of Cr correlate with a higher degree of authority. The Kendall concordance coefficient was employed to assess the level of agreement among the perspectives of these experts. A Kendall W value approaching 1, alongside a significance level of *P* < 0.05, suggests a higher degree of coordination among expert opinions.

##### 2.3.3.3 Pilot study

A pilot study was conducted to assess the accuracy and comprehensibility of the items in the initial scale. A convenience sample of 30 patients with coronary heart disease was recruited from a tertiary hospital in Hengyang to obtain feedback on the scale, following a straightforward formula for sample size calculation in pilot studies (31). The research team gathered questions and opinions based on the results and actual perceptions of the items, subsequently revising those that were unclear or ambiguous. A revised, user-friendly “cardiac rehabilitation adherence scale for patients with coronary heart disease” was developed through the modification, addition, and deletion of items.

#### 2.3.2 Phase2: the evaluation and exploration of the items

Item analysis was performed utilizing the subsequent analyses: Discrete tendency analysis: The discrete trend indicates the extent to which the values of each variable diverge from the central value. This study utilized the Coefficient of Variation (CV) to quantify the dispersion of each item. Items with a coefficient of variation less than 0.15 will be removed (32). 32 Critical ratio method: The scale's total scores were ranked in descending order. The upper group comprised the top 27% of individuals with the highest total scores, while the lower group consisted of the bottom 27% with the lowest total scores. An independent *t*-test was conducted to analyze the score differences for each item between the two groups. Items exhibiting a decision value (t value) exceeding 3, along with a statistically significant difference between the upper and lower groups (*p* < 0.05), were classified as strongly differentiated and subsequently retained (33). (3) correlation analysis: Pearson correlation analysis was performed to evaluate the applicability of the items. If the correlation coefficient between each item and the total score meets a minimum selection criterion of r ≥ 0.4, the item may be retained (34). The homogeneity test utilized Cronbach's *α* coefficient as the specific criterion. An increase in the Cronbach's *α* coefficient following the removal of an item suggests that the item may warrant elimination (35). The preliminary exploratory factor analysis was conducted to examine the factor loadings and assess the stability of the items. The minimum factor loadings recommended were 0.4, and cross-loadings were not allowed (36). If an item fails to meet the specified criteria, it is considered for removal from the pre-test scale.

#### 2.3.3 Phase3: the psychometric evaluation of the scale

According to the recommendation in the Consensus-based Standards for the Selection of Health Status Measurement Instruments (COSMIN) checklist (37), the psychometric properties of the cardiac rehabilitation adherence scale were evaluated, including content validity, structural validity and reliability.

##### 2.3.3.1 Content validity

Content validity refers to the degree to which questionnaire items accurately represent the concept that the researcher aims to measure (38). Content validity was evaluated using the content validity index (CVI), which encompasses the item-content validity index (I-CVI) for individual items and the scale-content validity index (S-CVI) for the overall scale. The I-CVI was ≥0.80 and the S-CVI was ≥0.90, indicating excellent content validity (39).

##### 2.3.3.2 Construct validity

Construct validity pertains to the degree of correlation between the measurement scale derived from survey results and the conceptual framework developed by the researcher (40). This scale's factor structure was analyzed through exploratory factor analysis (EFA) and confirmatory factor analysis (CFA). The sample was randomly partitioned into two groups: the EFA group (*n* = 254) and the CFA group (*n* = 255). An exploratory factor analysis using principal axis factoring was performed to investigate the underlying factor structure of the scale. The Kaiser-Meyer-Olkin (KMO) value exceeding 0.60, along with a statistically significant Bartlett's test of sphericity (*P* < 0.05), indicates the appropriateness of the data for exploratory factor analysis (EFA) (41). The specifications are outlined below: (1) The factor loading of the item is greater than or equal to 0.4, with no cross-loading present. (2) Each extracted common factor comprises at least three items. (3) All common factors must collectively account for more than 40% of the total variance (42). The CFA employed the maximum likelihood estimation method. The evaluation indices comprised the chi-square to degrees of freedom ratio (*χ*2/df), root mean square error of approximation (RMSEA), Tucker–Lewis index (TLI), incremental fit index (IFI), comparative fit index (CFI), goodness of fit index (GFI), parsimonious goodness of fit index (PGFI), and parsimonious normed fit index (PNFI). Model fit was deemed acceptable with the following criteria: *χ*2/df < 5, RMSEA < 0.08, and GFI, TLI, IFI, CFI > 0.90, along with PGFI and PNFI > 0.5 (43).

Additionally, the convergent and discriminant validity of the scale were evaluated to determine structural validity. The evaluation of convergent validity involved the calculation of the average variance extracted (AVE) and composite reliability (CR) values. A VE value exceeding 0.50 and a CR value surpassing 0.70 signify acceptable convergent validity of the scale (44). The square root of the AVE value and the correlation coefficients among factors were computed to assess discriminant validity. The square root of the AVE value must exceed the correlation coefficients among the corresponding factors (44).

##### 2.3.3.3 Reliability

Reliability denotes the consistency and stability of results obtained through research instruments (45). The reliability of the scale was assessed using Cronbach's *α* coefficient and the split-half reliability coefficient. The Cronbach's coefficient *α* was calculated for the total scale and each dimension. Cronbach's *α* coefficient values above 0.7 signify acceptable reliability (46). Split-half reliability was evaluated by determining the correlation between two segments of the scale, categorized into odd-numbered and even-numbered groups (47). A split-half reliability coefficient greater than 0.6 was deemed acceptable (47).

### 2.4 Data collection

In the initial phase, twenty-two qualified experts were provided with a condensed package that included informed consent and an expert consultation questionnaire via email, accompanied by a brief introduction outlining the study's purpose and significance. The experts were instructed to provide their feedback and suggestions within a two-week timeframe. In the second phase, 520 patients of the cardiology department in three hospitals were invited to participate in the survey, and all consented to the invitation and signed the informed consent form. Prior to participation, patients were informed regarding the study's purpose, significance, and the voluntary and anonymous nature of their involvement. The patients were required to complete and submit the questionnaires on site. During the completion of the questionnaires, team members conveyed interpretations of the questions and information through verbal communication, facilitating a clearer understanding of the items. The missing items were supplemented in time. The answering time of the data collection instrument was 15– 20 min for each participant.

### 2.5 Data analysis

SPSS (version 26.0) was utilized for correlation analysis, exploratory factor analysis, and reliability assessment, whereas AMOS 24.0 was applied for confirmatory factor analysis. Descriptive statistics were presented in terms of frequency and percentage. Content validity was assessed through item-level content validity index (I-CVI) and scale-level content validity index (S-CVI). Item analysis comprised the coefficient of variance (CV), critical value, and homogeneity tests. Maximum variance rotation was utilized in exploratory factor analysis to investigate the underlying factor structure. The structural equation model utilizing maximum likelihood was executed to assess the alignment between the underlying factor structure and theoretical expectations. The scale's reliability was assessed through Cronbach's *α* coefficient and the Spearman-Brown coefficient. A *p* value of less than 0.05 was considered statistically significant.

### 2.6 Ethics consideration

This study strictly adheres to the ethical principles outlined in the Declaration of Helsinki (48). Confidentiality and privacy of the participants were preserved. Ethic approval was gained from the Ethics Committee of the University (Approval no.2022112106). The purpose of the study and the procedure of participation were explained to the participants in detail. Participation was voluntary and all participants provided informed consent. Participants were informed of their right to withdraw from the study at any time.

## 3 Results

### 3.1 General information of participants

This study distributed 520 questionnaires, of which 509 were validly completed, resulting in an effective response rate of 97.88%, while 11 surveys had blanks and partial information. Among these, 48.92% were male and 51.08% were female. The predominant demographics of participants were those aged 50–70 (63.46%); married individuals (57.76%); those with education at junior high school level or lower (28.68%); employed persons (35.17%); and individuals with a monthly salary ranging from 3,000 to 5,000 yuan (34.77%). Table 2 presents the demographic profile of the participants (*N* = 509) involved in the study.

**Table 2:** Sociodemographic characteristics of participants (*N* = 509).

Item	Group	Number	Rate (%)
Gender	Male	249	48.92
	Female	260	51.08
Age	<50	80	15.72
	50∼	113	22.20
	60∼	130	25.54
	≥70	186	36.54
Education	Primary school or below	148	29.08
	Junior high school	146	28.68
	Senior high school	125	24.56
	College	54	10.61
	Bachelor degree or above	36	7.07
Marital status	Married	434	85.27
	Unmarried, divorced or widowed	75	14.73
Job status	civil servant	112	22.00
	Employee	179	35.17
	Farmer	143	28.09
	Others	75	14.73
Monthly income (RMB Yuan)	≦3,000	106	20.82
	3,001∼5,000	177	34.77
	5,001∼7,000	142	27.90
	7,001∼9,000	63	12.38
	>9,000	21	4.13

### 3.2 The generation and revision of the items

#### 3.2.1 Preliminary item pool

**Figure 2:**
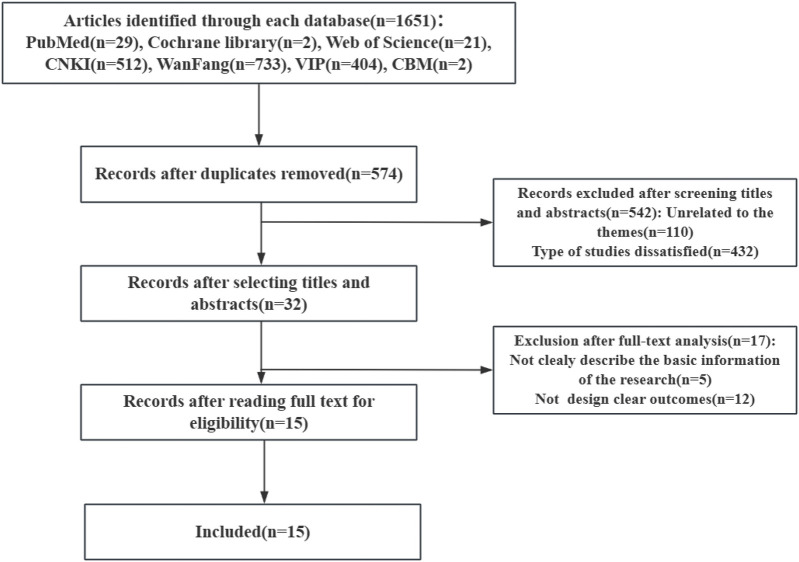
Flow diagram of literature review.

A total of 1,651 relevant articles were identified in the literature review, comprising 512 from CNKI, 404 from VIP, 733 from WanFang, 29 from PubMed, 2 from Cochrane Library, and 21 from Web of Science. Following the removal of duplicates and the sequential screening of titles and abstracts, 15 articles were selected for data analysis (Figure 2). Additionally, related guidelines such as the Guidelines for Cardiac Rehabilitation Programs by the American Association of Cardiovascular and Pulmonary Rehabilitation and the Guidelines for Cardiovascular Rehabilitation and Secondary Prevention in China were subsequently retrieved (49, 50). Outcomes reported in the reviewed literature were categorized into distinct domains. Following a team discussion regarding the practicability and feasibility of the items, five first-level indicators and thirty second-level indicators were developed based on five core perceptions: medication prescription, exercise prescription, nutrition prescription, psychological prescription, and smoking cessation for cardiac rehabilitation. The item pool comprises five dimensions assessing adherence to cardiac rehabilitation in patients with coronary heart disease (CHD), corresponding to five core perceptions in cardiac rehabilitation in China: exercise adherence (5 items), medication adherence (5 items), risk factor management adherence (8 items), nutrition management adherence (7 items), and psychology management adherence (5 items).

#### 3.2.2 Analysis of delphi survey and pilot study

Twenty-two eligible experts participated in two rounds of inquiry. The response rates of experts in both rounds were notably high, each achieving 100%. The substantial expert involvement underscores the importance of active participation in this study. The authority coefficients for the two rounds were 0.884 and 0.911, indicating that the expert panel possessed a strong familiarity with cardiac rehabilitation, thereby rendering their expert judgment reliable. The Kendall Wall values increased from 0.115 to 0.374 (*p* < 0.01), indicating improved concordance among experts regarding the items in the second round of inquiry. Following discussions with team members, the scale items were revised in accordance with the experts' recommendations. Nine items were added, and two items were removed. The initial version of the scale was established, comprising: exercise adherence (8 items), medication adherence (7 items), risk factor management adherence (8 items), nutrition management adherence (7 items), and psychology management adherence (5 items).

The pilot study included 30 participants from the researchers' hospital. Patients typically require 5–20 min to complete the questionnaire. Following feedback obtained from direct patient inquiries, no modifications were made to the content of the questionnaires. However, items within the exercise adherence dimension exhibited redundant expressions with analogous meanings. As a result, duplicate items were merged into one, improving the clarity and conciseness of the scale. A pre-test version of the scale comprising five dimensions and thirty-five items was developed.

### 3.3 The evaluation and exploration of the items

The item analysis revealed that the coefficient of variation for all items exceeded 0.15, while the critical ratio for the items varied from 1.93 to 46.85 (*P* < 0.05). The items-total correlation coefficients varied between 0.404 and 0.912 (*P* < 0.05), with the exception of item A7 (−0.812) and item D5 (0.281). The overall Cronbach's *α* coefficient for the scale was 0.909; however, the removal of items A7 and D5 would result in an increase in the coefficient. In the exploratory factor analysis, all items exhibited significant factor loadings between 0.415 and 0.915, with the exception of item A7 and item D5. Based on the results presented in Table 3, items A7 and D5 were excluded from the draft version, resulting in the development of a 33-item scale.

**Table 3:** Item analysis of the scale.

Item	Coefficient of variation	Critical ratio	Correlation coefficient	Cronbach's *α* coefficent	Retained item
A1	√	√	√	√	√
A2	√	√	√	√	√
A3	√	√	√	√	√
A4	√	√	√	√	√
A5	√	√	√	√	√
A6	√	√	√	√	√
A7	√	√	×	×	×
A8	√	√	√	√	√
B1	√	√	√	√	√
B2	√	√	√	√	√
B3	√	√	√	√	√
B4	√	√	√	√	√
B5	√	√	√	×	√
B6	√	√	√	√	√
B7	√	√	√	√	√
C1	√	√	√	√	√
C2	√	√	√	√	√
C3	√	√	√	√	√
C4	√	√	√	√	√
C5	√	√	√	√	√
C6	√	√	√	√	√
C7	√	√	√	√	√
C8	√	√	√	√	√
D1	√	√	√	√	√
D2	√	√	√	√	√
D3	√	√	√	√	√
D4	√	√	√	√	√
D5	√	√	×	×	×
D6	√	√	√	√	√
D7	√	√	√	√	√
E1	√	√	√	√	√
E2	√	√	√	√	√
E3	√	√	√	√	√
E4	√	√	√	√	√
E5	√	√	√	√	√

√means meeting the criteria,  ×  means not meeting the criteria.

### 3.4 The psychometric evaluation of the scale

#### 3.4.1 Content validity

The content validity of the scale was evaluated by a panel of 22 experts through two rounds of expert inquiry. The I-CVI ranged from 0.80 to 1.00 and the S-CVI was 0.96, indicating good content validity of the scale.

#### 3.4.2 Construct validity

##### 3.4.2.1 Exploratory factor analysis

**Figure 3:**
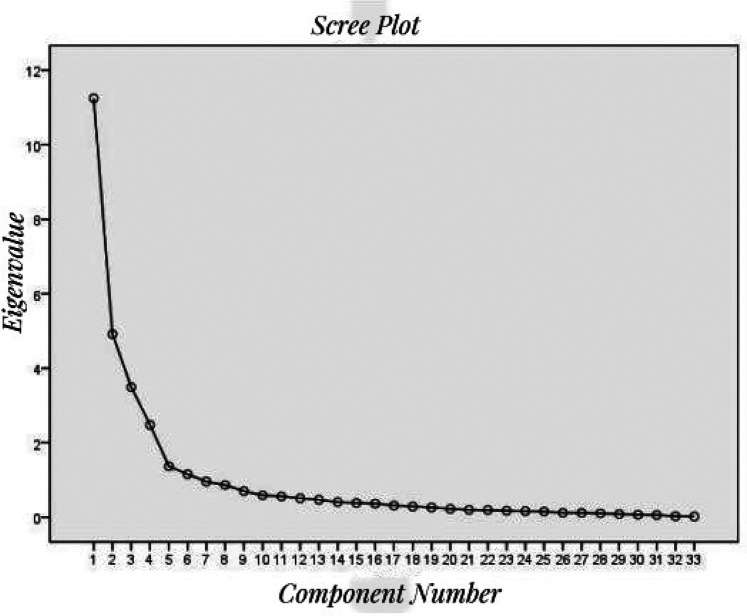
The scree plot of exploratory factor analysis.

Exploratory factor analysis (EFA) was employed to evaluate the structure of the scale. The KMO measure produced a high value of 0.861 (exceeding 0.80), and the Bartlett's test of sphericity was significant (*χ*2 = 16,406.328, *p* < 0.001), thereby confirming the suitability of the correlation matrix. A principal component analysis utilizing maximum variance rotation was conducted to identify common factors, yielding five factors with eigenvalues ≥1, which accounted for 71.255% of the total variation (Figure 3). Following varimax rotation, items with a factor loading of less than 0.4 were excluded. Consequently, items A7 and D5 were removed, while an additional 33 items were preserved due to their factor loadings exceeding 0.4. A rerun of principal component analysis was conducted on the remaining 33 items. The final exploratory factor analysis revealed a five-dimensional scale consisting of 33 items. The rotated factor loadings for the 33 items varied between 0.415 and 0.915 (Table 4). The five factors were identified: (1) exercise adherence (2) medication adherence (3) risk factor management adherence (4) nutrition management adherence (5) Psychology management adherence.

**Table 4:** Pattern matrix of the scale after the factor analysis.

Items	Components			
	1	2 3	4	5
A1	0.915			
A2	0.855			
A3	0.642			
A4	0.605			
A5	0.873			
A6	0.502			
A8	0.815			
B1		0.712		
B2		0.711		
B3		0.831		
B4		0.766		
B5		0.415		
B6		0.717		
B7		0.678		
C1		0.810		
C2		0.754		
C3		0.759		
C4		0.438		
C5		0.677		
C6		0.735		
C7		0.631		
C8		0.785		
D1			0.884	
D2			0.848	
D3			0.572	
D4			0.850	
D6			0.884	
D7			0.792	
E1				0.840
E2				0.901
E3				0.895
E4				0.881
E5				0.898

##### 3.4.2.2 Confirmatory factor analysis

**Figure 4:**
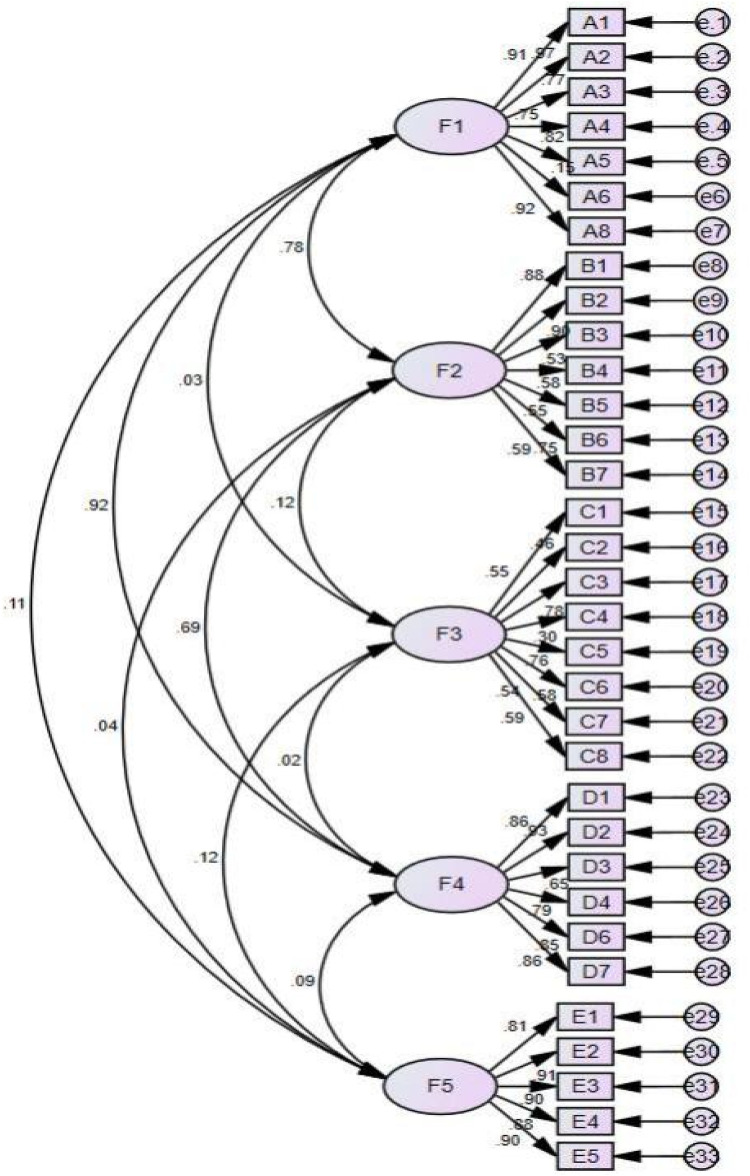
The five-factor model of cardiac rehabilitation scale (A1-E5 represent each scale item, while e1-e33 represent residuals).

The factor structure was assessed through confirmatory factor analysis (CFA) utilizing the maximum likelihood method, with data collected from 255 participants. The initial structure model in CFA underwent two modifications based on the modification index, and the revised fitting indicators were analyzed to determine the model's alignment with theoretical expectations (Figure 4). The fit indices were satisfactory, indicating a robust fit for the five-factor structure, with values of *χ*2/df = 1.774, RMSEA = 0.055, IFI = 0.931, TLI = 0.923, CFI = 0.930, PGFI = 0.718, and PNFI = 0.773, as presented in Table 5.

**Table 5:** Goodness-of-fit statistics of the scale.

Indices	Criteria	Result	Judgement
*χ*^2^/df	<5	1.774	Yes
RMSA	<0.08	0.055	Yes
IFI	≥0.9	0.931	Yes
TLI	≥0.9	0.923	Yes
CFI	≥0.9	0.930	Yes
PGFI	≥0.5	0.718	Yes
PNFI	≥0.5	0.773	Yes

##### 3.4.2.3 Convergent and discriminative validity

The assessment of convergent validity revealed AVE values between 0.516 and 0.798, and CR values ranging from 0.745 to 0.937 (Table 6), demonstrating satisfactory convergent validity. The analysis of discriminant validity revealed that the square root values of the AVE ranged from 0.649 to 0.893, each surpassing the correlation coefficients of their corresponding factors, indicating strong discriminative validity as shown in Table 7.

**Table 6:** The AVE values and CR values of the scale.

Indices	A	B	C	D	E
AVE	0.604	0.516	0.522	0.740	0.798
CR	0.937	0.745	0.906	0.754	0.845

**Table 7:** The correlation coefficient and sqrt (AVE) of the scale.

Factor	A	B	C	D	E
A	1.000				
B	0.719	1.000			
C	0.138	0.108	1.000		
D	0.752	0.684	0. 124	1.000	
E	−0.039	0.044	0.115	−0.059	1.000
Sqrt(AVE)	0.777	0.718	0.649	0.860	0.893

Sqrt (AVE) means the square root values of AVE.

### 3.5 Reliability analysis results

Table 8 presents a Cronbach's alpha coefficient of 0.909 for the total scale, with individual dimensions scoring 0.897, 0.842, 0.810, 0.931, and 0.937, respectively. These values indicate satisfactory internal consistency for the entire scale. The overall split-half reliability of the scale was 0.765, while the split-half reliability for each dimension was 0.903, 0.850, 0.841, 0.948, and 0.936, respectively, as presented in Table 8.

**Table 8:** Reliability tests: total scale and dimensions.

Scale	Cronbach's α coefficient	Spearman-Brown coefficient
Total scale	0.909	0.765
Exercise adherence	0.897	0.903
Medication adherence	0.842	0.850
Risk factor management adherence	0.810	0.841
Nutrition management adherence	0.931	0.948
Psychology management adherence	0.937	0.936

## 4 Discussion

The high global prevalence of coronary heart disease (CHD) underscores the necessity of developing a scale to assess adherence to cardiac rehabilitation (51). No research tools currently exist that measure cardiac rehabilitation adherence in patients with coronary heart disease (CHD). To address this gap, our team developed an evaluation tool focused on cardiac rehabilitation adherence in patients with coronary heart disease. The items were developed and refined based on an extensive literature review, expert consultation, and pilot testing. The initial scale comprised five dimensions: exercise adherence, medication adherence, risk factor management adherence, nutrition management adherence, and psychology management adherence. The five-factor structure of the scale was rigorously validated to assess its psychometric properties, addressing the limitations of previous tools and effectively measuring adherence to cardiac rehabilitation. This scale provides a reference for evaluating cardiac rehabilitation adherence and identifying the specific areas of weakness in patients' adherence, thereby enabling healthcare professionals to implement targeted interventions.

Factor 1 was designated as “exercise adherence” due to its inclusion of items that indicate the consistent and active participation of patients in cardiac rehabilitation. Exercise is an essential adherence behavior for patients with coronary heart disease in the context of cardiac rehabilitation (52). Exercise is fundamental to cardiac rehabilitation, supported by Level A1 evidence (53). Exercise is confirmed to play a crucial role in promoting cardiopulmonary function and improving clinical outcomes in cardiovascular diseases (54). Additionally, patients exhibiting high exercise adherence are able to quickly recognize exercise-related warnings and implement appropriate interventions to mitigate the risk of adverse cardiovascular events (55). It is crucial for patients to engage in cardiac rehabilitation exercise. Long-term adherence to cardiac rehabilitation exercise prescriptions poses significant challenges due to various obstacles (56). The telemonitored exercise rehabilitation models utilizing smartphones may partially mitigate these challenges through their intensity and flexibility (57). The exercise adherence dimension serves as a crucial component in evaluating cardiac rehabilitation adherence, providing a direct and accurate assessment of patients' adherence levels.

Factor 2 was designated as “medication adherence” due to its items assessing patients’ compliance with prescribed medications by healthcare professionals and their continuation of such medications. Medication adherence is a critical component of coronary heart disease management within the cardiac rehabilitation framework (58, 59). Multiple studies indicate that non-adherence and suboptimal adherence to medication correlate with adverse clinical outcomes in patients with coronary heart disease, including heightened mortality and rehospitalization rates (60, 61). Moreover, reports indicate that medication adherence among patients with a history of myocardial infarction varies between 13% and 61%, highlighting the significant challenge of achieving high adherence rates (62). Therefore, it is imperative for healthcare professionals to assist patients with CHD in improving their medication adherence. A systematic review demonstrated that educational programs can significantly enhance medication adherence in adults with coronary heart disease within two to six months following the intervention (63). Consequently, there is a significant need for high-quality and comprehensive educational programs in future research, particularly regarding the long-term effects following interventions.

Factor 3 was designated as “risk factor management adherence” due to its emphasis on promoting healthy lifestyles through the management of disease risk factors. Management of risk factors represents a crucial adherence behavior among individuals with coronary heart disease undergoing cardiac rehabilitation (64). Numerous risk factors contribute to coronary heart disease, including obesity, smoking, excessive alcohol consumption, and hypertension (65–68). A prior study indicated that around one-third of patients with acute coronary syndrome continued smoking or failed to adhere to health lifestyle recommendations (69). The self-management of disease risk factors presents significant challenges. Patients with coronary heart disease (CHD) face a significant risk of subsequent events and require rigorous management of risk factors (70). A randomized controlled trial demonstrated that one-way SMS text messages serve as a cost-effective adjunct for lifestyle modification aimed at preventing the recurrence of coronary heart disease (71). Utilizing mHealth tools, including interactive messages, personalized communications, and wearable devices, can enhance patients' self-management of risk factors.

Factor 4 was designated as “nutrition management adherence” due to its items concentrating on the daily dietary content for patients. Nutrition management is essential for patients' self-management in cardiac rehabilitation (72). Risk factors, including elevated blood sugar, high blood lipid levels, and obesity, are all associated with dietary habits. Nutrition management can mitigate risk factors and delay the progression of coronary heart disease (73, 74). The Mediterranean diet is characterized by low consumption of meat and meat products, particularly red meat and whole-fat dairy products, resulting in a relatively high-fat profile (75). This dietary pattern can enhance cardiovascular health by promoting beneficial alterations in blood fatty acid composition and lipoprotein levels, as well as providing protection against oxidative stress (76). A randomized intervention study has demonstrated that the Mediterranean diet is more effective than a control low-fat diet (77). Therefore, it is crucial for patients with coronary heart disease to adopt a Mediterranean diet in place of an unhealthy diet.

Factor 5 was designated as “psychology management adherence” due to its emphasis on assessing an individual's psychological condition. Effective psychology management is essential for improving patient outcomes in cardiac rehabilitation (78). Depression frequently occurs in patients diagnosed with coronary heart disease. A recent review indicates that around 40% of patients with coronary heart disease (CHD) also experience some form of depression, which negatively impacts health outcomes and prognosis (79). A systematic review indicates that anxiety is linked to a heightened risk of mortality in patients with coronary heart disease (CHD) (80). Therefore, it is essential that the care of individuals with CHD addresses both psychological and physiological needs. Xinkeshu tablets (XKS), a recognized Chinese patent medication, may enhance mental symptoms in patients with coronary heart disease following percutaneous coronary intervention (81). Future research should involve more rigorous multicenter studies with adequate sample sizes and double-blind randomized clinical trials to evaluate the effects of this drug.

The item analysis revealed that both the coefficient of variation and critical ratio surpassed the reference standard value, indicating adequate discrimination of the scale. With the exception of items A7 and D5, the correlation coefficients of the item-total scores exhibited moderate to high correlation, indicating that the scale demonstrates appropriate applicability. Furthermore, the removal of items A7 and D5 did not result in an increase in the Cronbach's coefficient, suggesting that the scale maintains appropriate homogeneity. Items A7 and D5 were excluded from the preliminary exploratory factor analysis. The factor loadings of the remaining items surpassed the recommended standard values, indicating greater stability. In conclusion, items A7 and D5 were removed, resulting in a total of 33 items for subsequent validation.

This study successfully confirmed both content validity and construct validity. The I-CVI and S-CVI exceeded the standard value, demonstrating the scale's adequate content validity (39). The scale's structural validity was assessed through an exploratory factor analysis (EFA) and subsequently a confirmatory factor analysis (CFA). The final EFA results indicate that the total variance explained by the five-factor structure was near the recommended threshold for multidimensional scales (42). This suggests that the scale effectively assesses patients' adherence levels in the cardiac rehabilitation process. The CFA results indicated a satisfactory model fit to the observed data, confirming the construct validity of the scale. The AVE and CR values were acceptable, and the square root of the AVE values exceeded the correlation coefficients between the corresponding factors, indicating that the scale demonstrates good convergent and discriminant validity. The cardiac rehabilitation scale is scientifically robust and exhibits strong construct validity. In this study, the internal consistency reliability of the final scale was confirmed. Both the Cronbach's *α* coefficients and the spilt-half reliability coefficients of the scale exceeded the recommended reference values, thus exhibiting an acceptable internal consistency (46, 47). Obeying the key principle of scale development strictly may be one of main reason why this scale gained satisfactory internal consistency reliability.

This study exhibited several notable strengths. This study followed a rigorous and transparent scale development process, ensuring the accuracy and applicability of the cardiac rehabilitation adherence scale. The scale thoroughly assesses patient adherence across five dimensions, consistent with guideline principles. This scale is more comprehensive than other tools, such as the cardiac rehabilitation adherence tool developed by Hamedani (82), as it encompasses a wider range of adherence behaviors related to cardiac rehabilitation. The task requires only 5–10 min for completion, making it straightforward to administer and relatively efficient. This study has several limitations that should be acknowledged. The participants were exclusively selected from inpatients at three tertiary hospitals, which restricted generalizability and introduced potential selection bias due to limited human resources. Secondly, the scale was developed and assessed within the framework of Chinese culture and population, which may restrict its cross-cultural applicability. To address these limitations, further research is essential to conduct among a broader range of patients in local clinics and other countries, thereby enhancing generalizability and applicability.

## 5 Conclusions

This study presents the development of a cardiac rehabilitation adherence scale and the validation of its psychometric properties in patients with coronary heart disease. The newly developed scale encompasses five dimensions and consists of 33 items. The tool effectively assesses the degree of adherence to cardiac rehabilitation among patients, exhibiting robust reliability and validity. Future research may utilize the developed scale by healthcare professionals to assess patient adherence levels and evaluate the impact of interventions, thereby identifying weaknesses and measuring effectiveness.

## Data Availability

The original contributions presented in the study are included in the article/Supplementary Material, further inquiries can be directed to the corresponding author.
