# Supplementary material for: Psychometric evaluation of the cardiac rehabilitation adherence scale in patients with coronary heart disease: an observational study
Source: Front Cardiovasc Med. 2025 Sep 11;12:1641392. doi: 10.3389/fcvm.2025.1641392 (PMC12460240; doi:10.3389/fcvm.2025.1641392)
Supplement: Supplementary file 1 [file Datasheet1.docx]

| **PubMed** | | |
| --- | --- | --- |
| #1 | "Coronary disease"[Mesh]) OR (coronary heart disease[Title/Abstract])) OR (myocardial ischemia [Title/Abstract])) OR (acute coronary syndrome  [Title/Abstract])) OR (myocardial infarction[Title/Abstract])) OR (percutaneous coronary intervention[Title/Abstract])) OR (percutaneous transluminal coronary  angioplast[Title/Abstract])) OR (“coronary artery bypass[Title/Abstract])) | 822784 |
| #2 | "Cardiac rehabilitation"[MeSH Terms] OR“Cardiac rehabilitations"[Title/Abstract]OR"Cardiovascular rehabilitation*"[Title/Abstract] OR "Rehabilitation *"[Title/Abstract] | 83659 |
| #3 | Adherence"[Title/Abstract] OR "compliance"[Title/Abstract] OR " persistence"[Title/Abstract] | 129653 |
| #4 | #1 AND #2 AND #3 | 29 |
| **Web of science（Core set）** | | |
| #1 | TS=( Coronary disease OR coronary heart disease OR myocardial ischemia OR acute coronary syndrome OR myocardial infarction OR percutaneous coronary intervention OR percutaneous transluminal coronary angioplast OR coronary artery bypass | 1121316 |
| #2 | TS=(Cardiac rehabilitation OR Cardiovascular rehabilitationOR " rehabilitation) | 371871 |
| #3 | TS=（Adherence* OR compliance* OR persistence* | 157414 |
| #4 | #1 AND #2 AND #3 | 21 |
| **Cochrane** | | |
| #1 | MeSH descriptor: [coronary disease] explode all trees | 134332 |
| #2 | MeSH descriptor: [coronary heart disease] explode all trees | 68389 |
| #3 | MeSH descriptor: [myocardial ischemia ] explode all trees | 27813 |
| #4 | MeSH descriptor: [acute coronary syndrome ] explode all trees | 3127 |
| #5 | (Coronary disease OR coronary heart disease OR myocardial ischemia OR acute coronary syndrome OR myocardial infarction OR percutaneous coronary intervention OR percutaneous transluminal coronary angioplast OR coronary artery bypass:ti,ab,kw | 156941 |
| #6 | #1 OR #2 OR #3 OR #4 OR #5 | 63935 |
| #7 | MeSH descriptor: [Cardiac rehabilitation] explode all trees | 81076 |
| #8 | MeSH descriptor: [rehabilitation] explode all trees | 132964 |
| #9 | MeSH descriptor: [cardiovascular rehabilitation] explode all trees | 56844 |
| #10 | (Cardiac rehabilitation OR Cardiovascular rehabilitationOR " rehabilitation):ti,ab,kw | 141538 |
| #11 | #7 OR #8 OR #9 OR #10 | 43482 |
| #12 | MeSH descriptor: [adherence] explode all trees | 79920 |
| #13 | MeSH descriptor: [compliance] explode all trees | 70064 |
| #14 | MeSH descriptor: [persistence] explode all trees | 30275 |
| #15 | (Adherence* OR compliance* OR persistence*):ti,ab,kw | 88188 |
| #16 | #12 OR #13 OR #14 OR #15 | 143276 |
| #17 | #6 AND #11 AND #17 AND #24 | 2 |
| **CNKI** | | |
|  | SU=冠心病+心脏病+心肌梗死+心肌缺血+AND SU= 心脏康复+康复+恢复+心血管康复AND SU=依从性+依赖性 | 512 |
| **WanFang** | | |
|  | 主题:(“冠心病+心脏病+心肌梗死+心肌缺血”) AND 主题:( “心脏康复+康复+恢复+心血管康复”) AND 主题:(“依从性+依赖性”) AND 主题:(“质性研究”+“扎根理论”+“现象学”+“描述性”+“行动研究”+“历史研究”+“民族志”+“人种学”+“个案研究”+“访谈”) | 733 |
| **Vip** | | |
|  | (M=(冠心病+心脏病+心肌梗死+心肌缺血 ) OR R=(心脏康复+康复+恢复+心血管康复)) AND (M=(依从性+依赖性) | 404 |
| CBM | | |
|  | 主题:(“冠心病+心脏病+心肌梗死+心肌缺血”) AND 主题:( “心脏康复+康复+恢复+心血管康复”) AND 主题:(“依从性+依赖性”) AND 主题:(“质性研究”+“扎根理论”+“现象学”+“描述性”+“行动研究”+“历史研究”+“民族志”+“人种学”+“个案研究”+“访谈”) | 2 |
